# Gradual withdrawal of remifentanil delays initial post-operative analgesic demand after thyroid surgery; double-blinded, randomized controlled trial

**DOI:** 10.1186/s12871-019-0731-9

**Published:** 2019-04-25

**Authors:** Sarah Saxena, Kimberly Gonsette, Willy Terram, Isabelle Huybrechts, Daniel A. Nahrwold, Matteo Cappello, Luc Barvais, Edgard Engelman

**Affiliations:** 1 Department of Anesthesia and Perioperative Care, CUB Erasme University Hospital, Université Libre de Bruxelles, 808, Route de Lennik, 1070 Brussels, Belgium; 2 Department of Anesthesia and Perioperative Care, Zuckerberg San Francisco General Hospital & Trauma Center, University of California, San Francisco, USA; 3 Department of Anesthesiology, H. Lee Moffitt Cancer Center & Research Institute, University of South Florida, Tampa, USA; 4 Department of Cardiothoracic Surgery, CUB Erasme University Hospital, Université Libre de Bruxelles, Brussels, Belgium

## Abstract

**Background:**

Mismanagement of remifentanil leads to severe side effects such as opioid-induced tolerance and hyperalgesia. Recently studies revealed an alternative withdrawal method to limit these side effects. A gradual withdrawal of remifentanil seems to be associated with less pain. The hypothesis of this double-blinded, randomized controlled trial was that a gradual withdrawal of remifentanil would be associated with less immediate post-operative pain compared to after an abrupt discontinuation of remifentanil in patients who underwent thyroid surgery.

**Methods:**

This double-blinded, randomized controlled trial was conducted in a tertiary level hospital in Brussels (Belgium) from April until August 2017. 34 patients undergoing thyroid surgery were randomized and 29 patients completed the study. After randomization, patients undergoing thyroid surgery were allocated to two groups: one with an abrupt discontinuation of remifentanil after surgery and one with a gradual withdrawal of remifentanil after surgery. The primary outcome was the initial post-operative demand of analgesic medication.

**Results:**

Gradual withdrawal of remifentanil was associated with a delayed initial post-operative demand of analgesic medication (*P* = 0.006). The first morphine bolus was given after 76.3 +/− 89.0 min in the group with a gradual withdrawal of remifentanil versus after 9.0 +/− 13.5 min in the group with an abrupt discontinuation of remifentanil.

However, overall morphine consumption, numeric rating scale scores, Ramsay Sedation Scale scores, and quality of recovery scores (QoR-40) were similar in both groups (*P* > 0.05).

**Conclusion:**

Though overall morphine consumption, numeric rating scale scores, Ramsay Sedation Scale scores, and quality of recovery scores (QoR-40) are not altered, a gradual withdrawal of remifentanil after thyroid surgery is safe and associated with a delayed initial post-operative demand of analgesic drugs. The withdrawal process does, however, require vigilance and training.

**Trial registration:**

Clinicaltrials.gov NCT03110653 (PI: Luc Barvais; date of registration: 03/31/2017).

## Background

Over the last decade, remifentanil has been subjected to bad press [1, 2]. Initially, this drug was hailed for its benefits which included being an ultra short-acting phenylpiperidine opioid analgesic with high lipid solubility, having a rapid onset of action, and being rapidly metabolized by non-specific blood and tissue esterases. For these reasons, many anesthesiologists became accustomed to using remifentanil. It can be given in high doses, is easily titratable, and leads to predictable, rapid recoveries.

However, the mismanagement of remifentanil can lead to a wide range of side effects including opioid-induced tolerance and hyperalgesia. Additionally, due to its rapid elimination, a bridge to post-operative analgesia is a necessity when administering this drug.

Opioid tolerance is defined as an increase in the dose required to maintain analgesia in patients receiving opioids for pain relief in the clinical setting. Opioid-induced hyperalgesia is a state of nociceptive sensitization and is defined as increased pain from a stimulus that normally provokes pain [3]. While clear definitions are accepted, the underlying mechanisms behind these concepts are still widely misunderstood and remain under investigation.

Recently, studies have been published about an alternative withdrawal method of remifentanil. Albrecht et al. demonstrated that immediate discontinuation of remifentanil after digestive surgery is associated with increased postoperative pain levels [4].

A gradual withdrawal of remifentanil may be associated with less pain in a rodent population [5]. Several research groups have evaluated this hypothesis in healthy human volunteers and have seemingly confirmed the theory that a gradual withdrawal of remifentanil is associated with less pain [6, 7].

Our group wanted to put this theory into practice within the perioperative period. The hypothesis of this double-blinded, randomized controlled trial was that a gradual withdrawal of remifentanil would be associated with less immediate post-operative pain compared to after an abrupt discontinuation of remifentanil in patients undergoing thyroid surgery.

## Methods

### Ethics

This study was designed adhering to the Declaration of Helsinki and the CONSORT checklist, and was approved by the internal review board (03/30/2017; P2017/074; Comité Ethique, Erasme hospital, Brussels; Chairman: Jean-Marie Boeymans). The trial was registered on clinicaltrials.gov (NCT03110653; PI: Luc Barvais; date of registration: 03/31/2017). Written informed consent was obtained from each patient participating in the study.

Inclusion criteria were the following: male or female patients aged 18–65 undergoing thyroid surgery at the Erasme hospital, Brussels, ASA physical status of I-III, and a knowledge of French, English, or Dutch.

Exclusion criteria were the following: pregnancy, hypo or hyperthyroidism, gastro-duodenal ulcer, allergy or contraindications to one of the study drugs, renal insufficiency, liver insufficiency, neuropsychiatric disturbance, BMI > 30, history of drug and alcohol abuse, and preoperative analgesic drug use.

The main objective of this study was to evaluate immediate post-operative pain levels in patients who underwent two types of remifentanil withdrawal methods. The timing of the first demand of post-operative analgesic was compared in both groups. In parallel, post-operative morphine consumption was also compared between both groups, as well as numeric rating scale (NRS) values at rest and after a small head flexion. In this way, pain was evaluated after 0, 15, 30, 45, 60, 75, 90, 105, 120 min, and 4 h after admission to the post-anesthesia care unit (PACU), as well as 24 h post-operatively. A quality of recovery (QoR-40) questionnaire was given to each patient pre- and post-operatively to evaluate patient satisfaction [8]. In the PACU, sedation was assessed in both groups via the Ramsay Sedation Scale [9].

Patients were randomized to one or the other group using a computer-generated randomization list (QuickCalcs program-GraphPad Software Inc., La Jolla, USA). For premedication, all patients received alprazolam (0.5 mg P.O.) one hour pre-operatively. Multi-parameter monitoring was used according to our institution’s protocol (ECG, pulse oximetry, non-invasive blood pressure every three minutes, Bispectral Index (BIS), and neuromuscular blockade monitoring utilizing the train-of-four ratio). In the case of blood pressure dropping 20% or more from initial baseline measurement, a bolus of intravenous ephedrine was administered.

Anesthesia was induced intravenously in both groups with remifentanil TCI (Minto model; 5 ng ml^− 1^; approximately 0.15 mcg kg^− 1^ min^− 1^) and propofol TCI (Schnider model; starting at 3 mcg ml^− 1^; adjusted to BIS levels between 45 and 55). Rocuronium (0.6 mg kg^− 1^) was then administered to facilitate tracheal intubation. After tracheal intubation, but pre-incision, remifentanil levels were lowered to 2 ng ml^− 1^ (approximately 0.065 mcg kg^− 1^ min^− 1^). Just before incision, remifentanil levels were increased to 5 ng ml^− 1^, and acetaminophen (1000 mg), diclofenac (1 mg kg^− 1^) and morphine (0.15 mg kg^− 1^) were administered as co-analgesics. Dexamethasone (10 mg) was administered to both groups. By the end of the surgery, ondansetron (4 mg) was given to all patients. Upon skin closure, remifentanil levels were progressively lowered to 3.5 ng ml^− 1^ (approximately 0.13 mcg kg^− 1^ min^− 1^). By the end of the surgery, propofol TCI was discontinued and remifentanil was kept at 2 ng ml^− 1^. Tracheal extubation was accomplished after adequate spontaneous ventilation was ensured, as well as the patient’s responsiveness to verbal commands.

The patients were then transferred to the PACU with the remifentanil TCI infusion pump running at 2 ng ml^− 1^. Back check valves and continuous saline infusion were used in the IV line in order to avoid backflow, dead space syndrome, or unintentional bolus.

According to pre-operative randomization, the remifentanil infusion syringe was then switched in the PACU to a new syringe, which was prepared by a colleague (WT) who was independent of the study. This post-operative syringe was either remifentanil diluted to 20 mcg/ml or NaCl 0.9%. PACU nurses and the primary anesthesiologist did not know whether their patients had received a newly prepared remifentanil or NaCl 0.9% syringe.

A gradual decrease of this infusion was then started in the PACU according to the following protocol: a reduction in infusion rate of 30% every 15 min (2 - > 1.4 - > 1 - > 0.7- > 0.5 - > 0.35 - > 0.25 - > 0 ng ml^− 1^). This gradual decrease was achieved in two hours.

While in the PACU, intravenous morphine (2 mg every five minutes) was administered if a patient’s pain was more than 3/10 on the NRS. All patients were monitored and under immediate medical surveillance, and therefore no limit was imposed on total morphine consumption. Additionally, acetaminophen (1000 mg every 6 h) and diclofenac (1 mg kg^− 1^ every 12 h) were administered to all patients. Patients stayed a minimum of four hours in the PACU and were discharged from the PACU once they were comfortable (e.g. NRS < 3/10, no post-operative nausea and vomiting, Ramsay scores of 2–3, no hematoma at the surgical site).

The total quantities of each drug used, as well as the different timings (induction, incision, end of surgery, and extubation times), were recorded. The primary anesthesiologist collected data pre-operatively and post-operatively (the first 24 h post-operation).

### Statistical analysis

As the primary outcome of the study was to compare the first post-operative demand of analgesic drugs, we considered a difference of 35 min or more to be of clinical relevance. With a two-sided alpha level of 0.05 and power of 90% (standard deviation (SD) of 27 min), the study needed to be conducted on 26 patients with each group consisting of 13 patients.

Statistix 9.0 (Analytical Software, Tallahassee, USA) was used for statistical analyses. The results were presented as mean ± standard deviation. *P* values ​​ < 0.05 were considered to be statistically significant. The Mann-Whitney U test was used for the analysis of the first demand of analgesic drug data, morphine consumption data, QoR-40 data, and NRS score data at 24 h. NRS values and Ramsay scale scores in the PACU were analyzed using a two-way ANOVA for repeated measures.

## Results

**Fig. 1:**
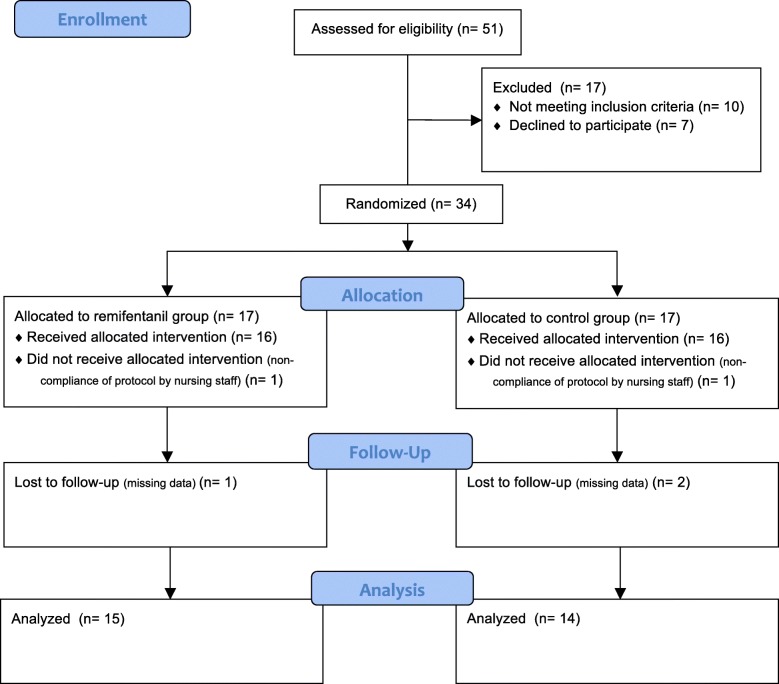
Flow diagram

Thirty-four patients were randomized from April to August 2017 (Fig. 1). Three patients were excluded from analysis due to missing data. Additionally, the protocol was not adhered to in two patients. Therefore, 29 patients were analyzed with 15 in the remifentanil group and 14 in the control group.

Patient characteristics are described in Table 1. Age, weight, BMI, gender, and ASA scores were collected for both groups pre-operatively. Our primary outcome measure was the timing of the initial demand of post-operative analgesic drug, and our data analysis revealed a statistically significant difference between the two groups (*P* = 0.006). The first morphine bolus was given 76.3 ± 89.0 min post-operatively in the remifentanil group and 9.0 ± 13.5 min in the control group (Table 2).

**Table 1:** Demographic data

	Remifentanil group	Control group	*P* value
Age (years)	42.0 ± 10.3	44.6 ± 12.2	0.50
Weight (kg)	72.5 ± 13.3	78.1 ± 20.5	0.56
BMI (kg/m^2^)	26.5 ± 4.7	27.2 ± 5.8	0.91
Gender (F/M)	14 / 1	12 / 2	0.60
ASA score (1/2)	2 / 13	0 / 14	0.48

Data as Mean ± SD or number of patients – Analysis by Mann-Whitney U test or Fisher exact test

**Table 2:** Initial demand of post-operative analgesic, morphine consumption, QoR-40 scores, NRS values (24 h)

	Remifentanil group	Control group	*P* value for difference between groups
Time to administration of the first iv morphine bolus (min)	76.3 ± 89.0 (45.0)	9.0 ± 13.5 (3.5)	0.006
Supplementary postoperative morphine during the first 4 post-operative hours (mg)	6.4 ± 5.1 (6.0)	8.3 ± 3.9 (8.0)	0.251
Supplementary morphine, including the administration at the beginning of surgery, during the first 4 post-operative hours (mg)	16.6 ± 4.9 (17.0)	18.0 ± 4.2 (18.5)	0.325
Numeric rating scale score for pain at rest 24 h after surgery	1.85 ± 1.46 (2.5)	0.91 ± 1.37 (0.0)	0.113
QoR-40 score	173.4 ± 16.3 (176.0)	179.0 ± 10.8 (178.5)	0.513

Data as Mean ± SD (median) - Analysis by Mann-Whitney U test

**Fig. 2:**
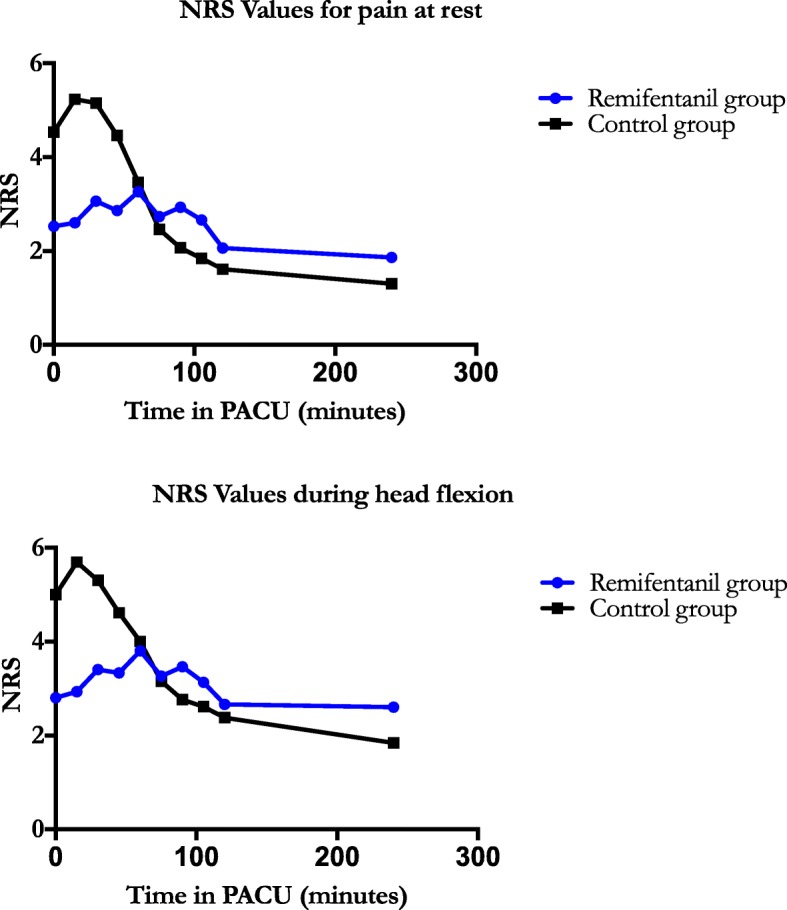
**a** Illustrates the Numeric Rating Scale (NRS) values in both groups during their stay in the PACU while at rest. Pain evaluation was done upon arrival to the PACU and every 15 min during the first 2 h post-operatively, as well as after 4 h. Patients evaluating their pain as > 3/10 on the NRS received a morphine bolus (2 mg every 5 min as needed). Values are Means ± SD. 2 way-ANOVA for repeated measures. *P* value for difference between both groups: 0.358; *P* value for difference between times: < 0.001; *P* value for interaction: < 0.001. **b** Illustrates the Numeric Rating Scale (NRS) values in both groups during their stay in the PACU during a light head flexion. Upon arrival and every 15 min during the first 2 h post-operatively, as well as after 4 h, patients were asked to do a light head flexion. During this moment, NRS values were recorded. Values are Means ± SD. 2 way-ANOVA for repeated measures. *P* value for difference between both groups: 0.418; *P* value for difference between times: < 0.001; *P* value for interaction: < 0.001

A statistically significant difference in morphine consumption was not observed while patients were in the PACU (*P* = 0.251). In addition, there was no difference in overall (intra- and post-operative) morphine consumption (*P* = 0.325; Table 2). Examination of NRS values in the PACU showed no statistically significant between-group differences both at rest (*P* = 0.358) and during head flexion (*P* = 0.418; Table 3; Fig. 2). Review of these same NRS values 24 h post-operatively on the ward did not demonstrate any statistically significant between-group differences (*p* = 0.113; Table 2). Furthermore, analysis of the QoR-40 scores exhibited no differences between the groups (*P* = 0.513; Table 2). Ramsay scores, indicating the patient’s sedation level, were not different between the groups (*P* = 0.337; Table 4).

**Table 3:** Numeric Rating Scale (NRS) Values for Pain

	PACU arrival	PACU + 15 min	PACU + 30 min	PACU + 45 min	PACU + 60 min	PACU + 75 min	PACU + 90 min	PACU + 105 min	PACU + 120 min	PACU + 240 min
NRS value for pain at rest										
Remifentanil group	2.53 ± 2.32	2.60 ± 2.19	3.06 ± 2.57	2.86 ± 1.99	3.26 ± 1.94	2.73 ± 1.71	2.93 ± 1.58	2.66 ± 1.54	2.06 ± 1.33	1.86 ± 1.72
Control group	4.53 ± 2.93	5.23 ± 2.86	5.15 ± 2.44	4.46 ± 2.22	3.46 ± 2.25	2.46 ± 1.94	2.07 ± 1.70	1.84 ± 1.34	1.61 ± 1.04	1.30 ± 1.25
	*P* value for difference between groups: 0.358 *P* value for difference between times: < 0.001 P value for interaction: < 0.001									
NRS value for pain during head flexion										
Remifentanil group	2.80 ± 2.33	2.93 ± 2.21	3.40 ± 2.66	3.33 ± 2.02	3.80 ± 1.93	3.26 ± 1.79	3.46 ± 1.50	3.13 ± 1.59	2.66 ± 1.75	2.60 ± 2.06
Control group	5.00 ± 3.13	5.69 ± 2.84	5.30 ± 2.84	4.61 ± 2.72	4.00 ± 2.76	3.15 ± 2.51	2.76 ± 2.42	2.61 ± 2.39	2.38 ± 2.32	1.84 ± 1.62
	P value for difference between groups: 0.418 *P* value for difference between times: < 0.001 *P* value for interaction: < 0.001									

Data as Mean ± SD - Analysis by 2-way ANOVA for repeated measures

**Table 4:** Ramsay Sedation Scale score

	PACU arrival	PACU + 15 min	PACU + 30 min	PACU + 45 min	PACU + 60 min	PACU + 75 min	PACU + 90 min	PACU + 105 min	PACU + 120 min	PACU + 240 min
Remifentanil group	2.46 ± 0.91	2.46 ± 0.64	2.53 ± 0.64	2.53 ± 0.64	2.40 ± 0.63	2.33 ± 0.61	2.33 ± 0.61	2.47 ± 0.74	2.47 ± 0.74	2.06 ± 0.25
Control group	2.21 ± 0.57	2.07 ± 0.47	2.07 ± 0.47	2.14 ± 0.36	2.14 ± 0.36	2.37 ± 0.63	2.28 ± 0.46	2.42 ± 0.75	2.35 ± 0.74	2.21 ± 0.57
	*P* value for difference between groups: 0.337 *P* value for difference between times: 0.224 *P* value for interaction: 0.047									

Data as Mean ± SD - Analysis by 2-way ANOVA for repeated measures

## Discussion

This double-blinded, randomized controlled trial showed that a gradual decrease of remifentanil levels is associated with a delayed initial demand of post-operative analgesic drug. This corresponds with what others have already described in healthy volunteers [6, 7].

Remifentanil-induced hyperalgesia remains a misunderstood concept, with multiple theories attempting to find its cause. One interesting cellular model for pain amplification and hyperalgesia after opioid withdrawal is referred to as the long-term potentiation (LTP) of synaptic strength in nociceptive pathways. Opioid withdrawal LTP has been described as the following: “A brief application of remifentanil *in vivo* leads to acute depression of synaptic strength in C-fibers. Upon withdrawal, synaptic strength not only quickly returns to normal, but becomes potentiated for prolonged periods of time” [10]. A study on spinal dorsal horns from rats showed that withdrawal LTP may be prevented by tapering of the remifentanil infusion instead of abrupt withdrawal [5]. This LTP could indeed explain why a gradual withdrawal of remifentanil was associated with a delayed initial post-operative analgesic demand.

Another theory explaining remifentanil-induced hyperalgesia is the activation of the N-methyl-D-aspartate (NMDA) receptor, which counteracts its analgesic effects. Indeed, blocking NMDA receptors can prevent this development by decreasing the activation of pronociceptive systems that are triggered by opioids [11].

In our study, remifentanil was administered in conjunction with propofol. Propofol, by inhibiting the NMDA subtype of the glutamate receptor, may have a preventative effect on remifentanil-induced hyperalgesia [1, 12].

The administration mode of remifentanil was based on the Minto model, which is relatively standard for most European countries. A TCI mode of remifentanil seems to be associated with less hyperalgesia [13].

The analgesia protocol of this trial was designed to prevent post-operative pain as much as possible [3]. A multimodal analgesia regimen (consisting of NSAIDS, acetaminophen, and timely administered morphine) is standard practice in our institution and was therefore applied to this study. This allowed us to keep remifentanil levels relatively low (at maximum 5 ng ml^− 1^ or approximately 0.15 mcg kg^− 1^ ml^− 1^).

In this study, remifentanil was gradually withdrawn in the PACU over the course of two hours, though at present, no consensus exists regarding the exact withdrawal method of remifentanil.

As mentioned, mismanagement of remifentanil administration leads to severe side effects. This study was conducted with extreme caution and required training of the nursing and anesthesia staff in order to avoid accidental boluses, especially during transport from the operating room to the PACU. In this way, episodes of respiratory depression and apnea were nonexistent (SP02 remained > 95% at all times). Sedation levels were the same in both groups, and all patients underwent anesthesia and the subsequent 24 h post-operative period uneventfully.

### Limitations

A limitation of our study was that hyperalgesia was not properly assessed through pain threshold tests at the surgical site. Pain was evaluated through the NRS, total morphine consumption, and initial post-operative demand of an analgesic.

Even though this initial demand of an analgesic was delayed through a gradual withdrawal of remifentanil, overall morphine consumption was quite low and equal in both groups (16.6 ± 4.9 mg in the remifentanil group versus 18.0 ± 4.2 mg in the control group). This could be explained by the fact that a well-timed multimodal analgesia plan was applied to both groups. Additionally, our study may not have been powered to show a difference in morphine consumption.

Though the difference in immediate post-operative NRS values between both groups was not statistically significant, some interesting observations can be made. While both groups benefitted from the same perioperative multimodal analgesia strategy, raw NRS values showed a remarkably different trajectory (Fig. 2). The control group (*n* = 14) had declining NRS values starting relatively high, whereas the remifentanil group (*n* = 15) had zigzag values centered around 3/10. A likely explanation for this is that the remifentanil group still received an opioid, remifentanil, in addition to morphine, and was therefore less prone to experiencing pain. However, the remifentanil concentrations were quite low, especially in the second hour of the withdrawal phase, and consequently may not completely explain the observed differences in NRS values. Again, it could also be that our study was not powered adequately to fully examine NRS values.

Though the study demonstrated a delayed initial post-operative analgesic demand after a gradual withdrawal of remifentanil, another limitation was that it was conducted on patients who underwent relatively minor thyroid surgeries. Larger studies must be done in major surgeries in order to see whether the results from this study can be reproduced in patients who experience greater noxious stimuli and further our understanding of some of the NRS interactions revealed in our study.

## Conclusion

Though overall morphine consumption, numeric rating scale scores, Ramsay Sedation Scale scores, and quality of recovery scores (QoR-40) are not altered, a gradual withdrawal of remifentanil after thyroid surgery is safe and associated with a delayed initial post-operative demand of analgesic drugs. The withdrawal process does, however, require vigilance and training.

## Abbrevations

LTP: Long term potentiation
NMDA: N-methyl-D-aspartate
NRS: Numeric Rating Scale
PACU: Post-anesthesia care unit
QoR: quality of recovery
TCI: Target controlled infusion

## References

[1] Fletcher D, Martinez V (2014). Opioid induced hyperalgesia in patients after surgery: a systematic review and a meta-analysis. Br J Anaesth.

[2] Guignard B, Bossard AE, Coste C (2000). Acute opioid tolerance: intraoperative remifentanil increases postoperative pain and morphine requirement. Anesthesiology.

[3] Yu EHY, Tran HD, Lam SW, Irwin MG (2016). Remifentanil tolerance and hyperalgesia: short-term gain, long term pain?. Anaesthesia.

[4] Albrecht S, Fechner J, Geisslinger G (2000). Postoperative pain control following remifentanil-based anaesthesia for major abdominal surgery. Anaesthesia.

[5] Didla R, Gassner M, Gingl E, Sandkuhler J (2009). Induction of synaptic long-term potentiation after opioid withdrawal. Science.

[6] Comelon M, Raeder J, Stubhaug A, Nielsen CS, Draegni T, Lenz H (2016). Gradual withdrawal of remifentanil infusion may prevent opioid induced hyperalgesia. Br J Anaesth.

[7] Sprenger C, Eichler IC, Eichler L, Zollner C, Buchel C (2018). Altered signaling in the descending pain modulatory system after short-term infusion of the mu-opioid agonist remifentanil. J Neurosci.

[8] Myles PS, Weitkamp B, Jones K, Melick J, Hensen S (2000). Validity and reliability of a postoperative quality of recovery score: the QoR-40. Br J Anaesth.

[9] Ramsay MA, Savege TM, Simpson BR, Goodwin R (1974). Controlled sedation with alphaxolone-alphadalone. BMJ.

[10] Sandkühler J, Gruber-Schoffnegger D (2012). Hyperalgesia by synaptic long-term potentiation (LTP): an update. Curr Opin Pharmacol.

[11] Richebé P, Cahana A, Rivat C (2012). Tolerance and opioid-induced hyperalgesia. Is a divorce imminent. Pain.

[12] Lee M, Silverman SM, Hansen H, Patel VB, Manchikanti L (2011). A comprehensive review of opioid-induced hyperalgesia. Pain Physician.

[13] Richebé P, Pouquet O, Jelacic S (2011). Target-controlled dosing of remifentanil during cardiac surgery reduces postoperative hyperalgesia. J Cardiothorac Vasc Anesth.

